# RTE and CTE mRNA export elements synergistically increase expression of unstable, Rev-dependent HIV and SIV mRNAs

**DOI:** 10.1186/1742-4690-3-6

**Published:** 2006-01-13

**Authors:** Sergey Smulevitch, Jenifer Bear, Candido Alicea, Margherita Rosati, Rashmi Jalah, Andrei S Zolotukhin, Agneta von Gegerfelt, Daniel Michalowski, Christoph Moroni, George N Pavlakis, Barbara K Felber

**Affiliations:** 1 Human Retrovirus Pathogenesis Section, National Cancer Institute-Frederick, Frederick, MD 21702-1201, USA; 2 Human Retrovirus Section, National Cancer Institute-Frederick, Frederick, MD 21702-1201, USA; 3 Institut für Medizinische Mikrobiologie Universitaet Basel, Basel, Switzerland

## Abstract

Studies of retroviral mRNA export identified two distinct RNA export elements utilizing conserved eukaryotic mRNA export mechanism(s), namely the Constitutive Transport Element (CTE) and the RNA Transport Element (RTE). Although RTE and CTE are potent in nucleocytoplasmic mRNA transport and expression, neither element is as powerful as the Rev-RRE posttranscriptional control. Here, we found that whereas CTE and the up-regulatory mutant RTEm26 alone increase expression from a subgenomic *gag *and *env *clones, the combination of these elements led to a several hundred-fold, synergistic increase. The use of the RTEm26-CTE combination is a simple way to increase expression of poorly expressed retroviral genes to levels otherwise only achieved via more cumbersome RNA optimization. The potent RTEm26-CTE element could be useful in lentiviral gene therapy vectors, DNA-based vaccine vectors, and gene transfer studies of other poorly expressed genes.

## Background

Posttranscriptional events determine the fate of cellular and viral mRNAs through concerted actions promoting nuclear trafficking and cytoplasmic transport, stabilization and translation. Simian type D (SRV/D) retroviruses and intracisternal A-particle retroelements (IAP) have provided us with unique mRNA transport elements, which utilize conserved cellular export machinery [1-13]. The export of the SRV/D unspliced mRNA is mediated by the cis-acting constitutive transport element CTE [8,10-13] through interaction with the cellular NXF1 protein [1], which is also the key factor mediating general mRNA export [1-5], a property which is conserved among eukaryotes (reviewed in [14-16]). We previously identified another functionally similar but structurally unrelated posttranscriptional RNA Transport Element RTE [6,7], which is present in a subgroup of murine IAP. Both CTE and RTE utilize the conserved eukaryotic mRNA transport machinery. Here, we demonstrate that the combination of RTE and CTE *in cis *leads to synergistic increase in lentiviral gene expression.

## Results

### Synergistic activation of gene expression in the presence of a combination of RTE-CTE

**Figure 1:**
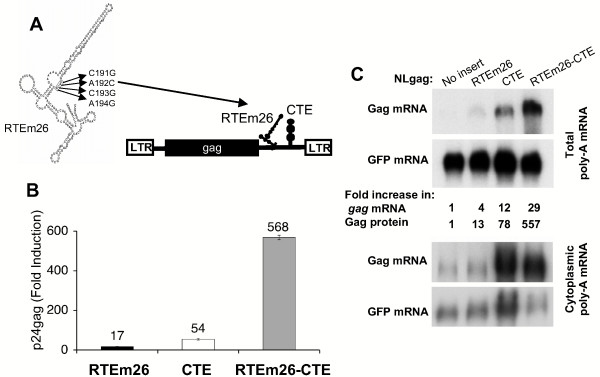
**RTEm26-CTE is a potent combination of RNA transport elements**. A) Structure of the *gag *reporter plasmid. The HIV-1 *gag *gene is flanked by the 5' and 3'LTRs providing promoter and polyadenylation signals, respectively. NLgag contains the major splice donor of HIV-1 located 5' to *gag *and a cryptic splice acceptor between RNA export elements and the 3'LTR and expresses HIV-1 gag [23, 24, 39]. The RTE structure [7] shows the nucleotide changes in mutant RTEm26 (nt 190–193 CACA changed to GCGG). The 226-nt RTE and the 173-nt CTE were inserted between the *gag *gene and the 3'LTR, generating the NLgagRTEm26-CTE. B) Expression of the *gag *reporter pNLgag plasmids, containing either no insert, RTEm26 or CTE alone, or the RTEm26-CTE combination. Cell extracts from transfected HeLa cells were analyzed for Gag production using an HIV-1 gag antigen capture assay. Gag expression is presented as fold induction as compared to the gag levels produced by pNLgag. Standard deviations are shown. C) Northern blots of total polyA-containing (top panel) and cytoplasmic (bottom panel) mRNAs from cells transfected with pNLgag or pNLgag containing RTEm26, CTE, or RTEm26-CTE were hybridized with a probe spanning the 3'end of the gag mRNAs [12]. Hybridization of the blot with a GFP probe serves as internal control of transfection efficiency and RNA preparation. The blots shown in the top and bottom panels are from two independent experiments. Note that the cytoplasmic poly-A mRNA samples are unequally loaded, and the CTE lane has 2.5-fold more GFP mRNA while the RTEm26-CTE lane has 60% of the GFP mRNA compared to the other lanes (no insert, RTEm26). The blots were quantitated using the STORM860 phosphoimager.

**Figure 2:**
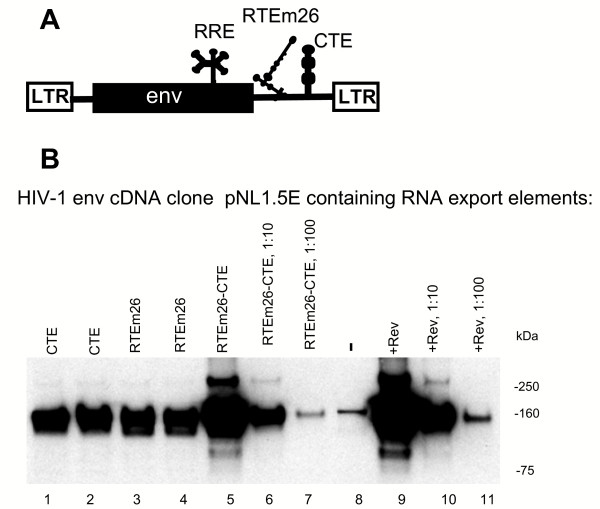
**RTEm26-CTE synergistically increase HIV-1 *env *production**. A) The structure of the *env *cDNA plasmid pNL1.5E containing the RTEm26-CTE. The *env *gene contains the Rev-responsive element RRE within *env *and is expressed from the HIV-1 LTR promoter. RTE, CTE and RTEm26-CTE were inserted between the *env *gene and the 3' LTR. B) HLtat cells were transfected with the indicated plasmids and analyzed for Env production by Western blot analysis using a rabbit anti-HIV-1 env serum.

Since the presence of RTE or CTE positively affects production of poorly expressed retroviral genes, we asked whether the RTE-CTE combination *in cis *has an additive or synergistic effect on gene expression. For this, we used the up-regulatory mutant RTE (RTEm26) (Figure 1A), known to increase RTE function by 2-fold [7], in combination with the SRV-1 CTE. The reporter plasmids used for these studies encode HIV-1 *gag *or *env *genes (Figures 1 and 2), which are known to be poorly expressed in the absence of a positive-acting posttranscriptional regulatory system [17-29]. In pNLgagRTEm26-CTE, the RTEm26 was inserted 5' to the CTE into reporter pNLgagCTE (Figure 1A). Upon transfection into human HeLa cells, we found that whereas RTEm26 or CTE alone activated Gag production by ~20-fold and ~50-fold, respectively (Figure 1B) as expected, the combination of these elements had a synergistic effect, leading to a dramatic ~570-fold activation (Figure 1B). Synergy was only observed when the elements were present in *cis*, but not upon co-transfection of the RTE- and CTE-containing reporters within the same cells (data not shown). Similar data were obtained by using a splice donor-deleted *gag *reporter, pNLcgag [24], which only produces an unspliced gag mRNA [24]. This experiment suggests that the synergistic effect of RTEm26-CTE is independent of splicing (data not shown). Analysis of total poly-A containing mRNAs from the transfected HeLa cells (Figure 1C) showed that the presence of either element alone elevated *gag *mRNA levels (4- and 12-fold, respectively) and the RTEm26-CTE combination resulted in a further increase (29-fold). Analysis of cytoplasmic mRNA (Figure 1C, bottom panel) confirmed that RTEm26-CTE promotes an increase of the cytoplasmic level of the reporter *gag *mRNA that is in accord with elevated levels of Gag protein production. We also noted a reproducible difference in the increase of *gag *mRNA and Gag protein levels, suggesting that posttranscriptional regulation was affected at all steps from transport, stabilization to translation. This is in accord with previous observations [30-33] that posttranscriptional regulation of such mRNAs includes both export and translation.

### Synergistic effect of RTEm26CTE on HIV-1 env expression

To rule out that the observed synergistic effect is a unique feature of the *gag *reporter mRNA, we inserted RTEm26-CTE into an HIV-1 *env *reporter plasmid pNL1.5E (Figure 2A), expressing the authentic *env *cDNA from the HIV-1 LTR promoter. Like *gag*, *env *is poorly expressed (Figure 2B, lane 8) in the absence of a positive-acting export system, as expected. Both plasmids, containing either CTE (lanes 1, 2) or RTE (lanes 3, 4) alone, showed ~10× fold increase in Env production compared to the pNL1.5E (lane 8). The presence of RTEm26-CTE led to an additional increase in Env production (lane 5). A semi-quantitative analysis using serial dilutions (lanes 5–7) of the cell extract shows a ~100× fold activation, confirming synergistic effect of RTEm26-CTE. This expression level was comparable to that obtained in the presence of Rev (lanes 9–11). These data demonstrate that the synergistic effect of the combination of RTEm26-CTE export elements is applicable for different poorly expressed, unstable HIV-1 mRNAs.

### Synergistic effect of RTEm26CTE on expression of a Rev- and RRE-deficient HIV-1 and SIV molecular clones

**Figure 3:**
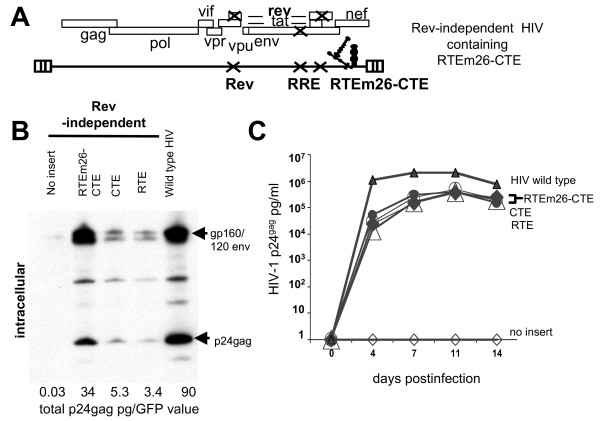
**RTEm26-CTE replaces Rev/RRE of HIV**. A) Structure of *rev *and RRE-minus HIV-1 containing RTEm26-CTE. Multiple point mutations inactivate both rev and RRE. CTE, RTE or RTEm26-CTE were inserted between env and the 3'LTR, rendering these clones nef-minus. B) Human 293 cells were transiently transfected with the indicated plasmids. Two days later, cell extracts were analyzed on Western immunoblots using HIV patient sera. Total intra- and extracellular Gag production was measured using commercial HIV p24 antigen capture assays and GFP production was quantitated. Normalized values (total gag in pg/total GFP units) are shown. C) HIV propagation in Jurkat cells. Transfected 293 cells were cocultivated with Jurkat cells: wild type NL4-3 (filled triangle), the Rev-independent HIV containing RTEm26-CTE (two clones filled diamond, open circle), CTE (open triangle), RTE (filled circle), and no insert (open diamond). Virus production was monitored over time using a commercial HIV p24gag antigen capture assay. Similarly, upon cell-free infection (not shown), the RTEm26-CTE replicates to a similar extent like the RTE- or CTE-containing Rev-independent HIV viruses.

**Figure 4:**
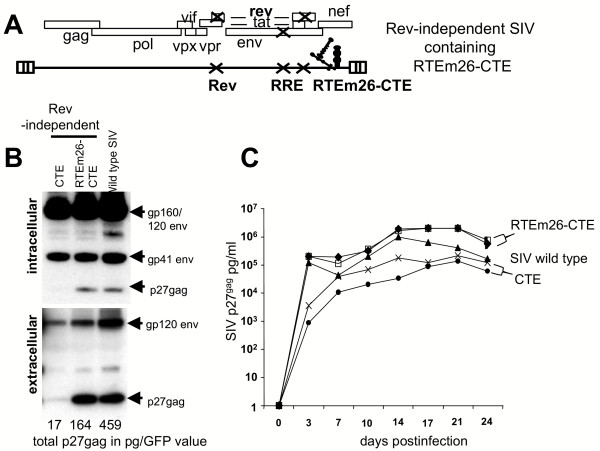
**RTEm26-CTE replaces rev/RRE of SIV**. A) Structure of the *rev*- and RRE-minus SIVmac239 containing RTEm26-CTE. Multiple point mutations inactivate both *rev *and RRE of SIVmac239. CTE or RTEm26-CTE was inserted between *env *and the 3'LTR. B) Human 293 cells were transiently transfected with the indicated plasmids. Two days later, cells and supernatant were analyzed for *gag *and *env *expression. Intracellular (1/10 of cell extract) and extracellular (1/150 of supernatant) were analyzed on Western immunoblots using a serum pool from SIV-infected monkeys. Total intra- and extracellular Gag production was measured using commercial SIV p27gag antigen capture assay and GFP production was quantitated. Normalized values (total gag in pg/total GFP units) are shown. C) SIV propagation in CEMx174 cells. Virus stock were generated upon cocultivation of transfected 293 cells with CEMx174 cells and then used to infect fresh CEMx174 cells: wild type SIVmac239 (filled triangle), two stocks containing the Rev-independent SIV containing CTE (filled circle and X, respectively), and two stocks containing the RTEm26-CTE (filled diamond and open square, respectively). Virus production was monitored over time using a commercial SIV p27gag antigen capture assay.

To test the synergistic potency of the RTEm26-CTE in a more complex system, we inserted the combination element into the Rev- and RRE-minus molecular clones of HIV-1 NL4-3 (Figure 3) and SIVmac239 (Figure 4). Both of these viruses are unable to produce structural proteins or infectious virus in the absence of the viral Rev/RRE regulatory system [6,11,12,19,23,34,35] (see also Figure 3B).

Upon insertion of CTE or RTE alone into the Rev- and RRE-minus NL4-3, we had previously shown that these RNA transport elements are able to partially replace the viral Rev-RRE system resulting in the production of infectious virus [6,9,11,12] (Figure 3B and 3C). Interestingly, Western immunoblot analysis showed that the presence of RTEm26-CTE mediated a dramatic synergistic increase in expression of both *env *and *gag *compared to the clones containing each element alone (Figure 3B). Quantitation of *gag *expression using an antigen capture assay showed an increase of ~1 log over the presence of CTE or RTE alone. The expression level in the presence of RTEm26-CTE was only slightly lower (~3x-fold) than those obtained by the wild type HIV-1 NL4-3 (Figure 3B). Upon infection of Jurkat cells, the RTEm26-CTE containing Rev-independent HIV-1 clone as well as the RTE- or CTE-containing clones showed similar replicative capacities to levels ~1 log lower than that of the wild type HIV-1 (Figure 3C). Thus, the presence of RTEm26-CTE is able to promote a balanced expression of the viral proteins able to generate infectious virus.

Similarly, we found that the presence of RTEm26-CTE also greatly increased expression of the Rev- and RRE-minus molecular clone of SIVmac239 (Figure 4B) to levels about ~10x-fold higher than those obtained by SIV clone containing only the CTE. Like its HIV counterpart, the RTEm26-CTE-containing SIV produces infectious virus (Figure 4C). We noted that it replicates with growth kinetics similar to the wild type SIV, in both CEMx174 cells (Figure 4C) and monkey PBMCs (data not shown), despite its slightly reduced level of expression (Figure 4B). In contrast to its HIV counterpart, the presence of the more potent RNA export element combination improved the replicative capacity when compared to the virus that contains only the CTE (compare peak at day 14 postinfection). Since we could not test propagation of SIV and HIV in the same cell types, it is possible that cellular factors may contribute to this phenomenon and this was not further investigated.

In conclusion, we have shown that the potent posttranscriptional effect of the RTEm26-CTE combination of RNA export elements from simple expression vectors (Figures 1 and 2) as well as from the complex array of mRNAs produced from the molecular clones of HIV and SIV (Figures 3 and 4).

### Synergy depends on the spatial arrangement of RTEm26 and CTE

**Figure 5:**
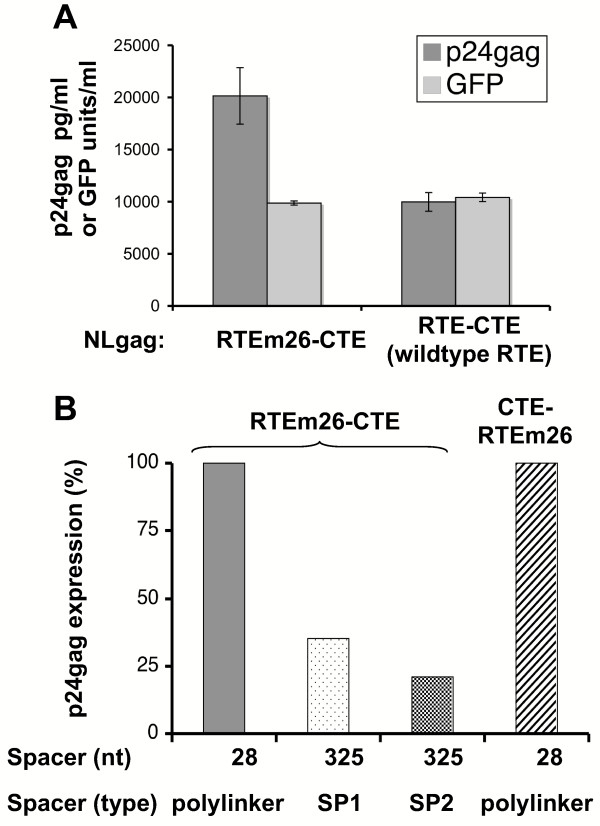
**Optimal design of RTEm26-CTE combination element**. A) Expression of pNLgag containing up-regulatory mutant RTEM26 or the wild type RTE in combination with the CTE. HeLa cells were transfected with the independent clones of indicated plasmids and analyzed for Gag expression as described in Figure 1. Standard deviations are shown. B) Organization of RTEm26-CTE element. pNLgag containing either RTEm26-CTE or the CTE-RTEm26, having the elements in reverse order separated by a 28-nt polylinker spacer, were analyzed. A spacer of 325 nt from either a synthetic HIV-1 *tat *gene (SP1) or from the *cat *gene (SP2) were inserted between RTEm26 and CTE in pNLgagRTEm26-CTE. A typical experiment is shown using the average of two to four plasmids per construct. The data are presented in % of Gag production by normalizing the values produced by pNLgagRTEm26-CTE to 100%.

To further understand the mechanism of the synergistic effect we generated a series of RTE-CTE containing plasmids with variations in the type of elements and their spatial arrangement. Since all our expression vectors utilize the 3' LTR as polyadenylation signal, we first asked whether the choice of this signal could contribute to the synergistic effect. Replacing the HIV-1 polyadenylation signal with that of the bovine growth hormone had no effect (data not shown). Next, we tested the effect of wild type RTE instead of the up-regulatory mutant RTEm26. Figure 5A shows that the substitution of RTEm26 within the context of the combination element with the wild type RTE led to a ~2-fold lower expression. This reduction can be explained by the 50% reduced activity of the wild type RTE compared to RTEm26 [7]. To further support the notion that active elements are required for synergy, we tested the combination of RTEm26 and the inactive CTE (mutant CTEm36 [8]), which lacks the NXF1 binding site but maintains the overall secondary structure. This combination of elements showed activity similar to a single RTEm26 (data not shown). Therefore, to achieve maximal synergistic effect requires the presence of both elements in their most active form.

We then tested whether the spatial arrangement of RTEm26 and CTE contributed to the synergistic effect. First, the reversal of the order of the elements from RTEm26-CTE to CTE-RTEm26 (Figure 5B) showed that the combination of the elements functions similarly in either configuration. Second, the 28 nt spacer between the elements was increased by insertion of a 325-nt spacer sequence (SP1), which led to a significant loss of synergy (Figure 5B). To exclude that the nature of the spacer RNA contributed to this effect, a different RNA fragment (SP2) was used (Figure 5B), resulting in a similar decrease in *gag *expression. Separation of the elements by shorter spacers of 202 and 100 nt led to gradual decrease in RTEm26-CTE activity (data not shown). Thus, the optimal synergistic effect requires the up-regulatory mutant RTE (RTEm26) and a functional CTE at close proximity.

The question arose whether multimers of CTE or RTE alone could achieve a similar effect. The presence of a CTE multimer has been reported to improve expression i.e. 4xCTE in a *gag*/*pol *reporter further elevated expression but this depended on the nature of the polyA signal [36], whereas multiple copies of the CTE had little or no effect in other mRNAs ([36], our own observations), suggesting that an effect of CTE multimers depended on the mRNA context. Using the *gag *reporter plasmid used herein, pNLgag, we found that two adjacent CTE elements also synergized reaching ~75% of the effect observed for RTEm26-CTE. In contrast, we found that RTEm26 does not synergize with itself. Thus, while the effect of CTE multimers is transcript dependent, the RTEm26-CTE mediated synergistic increase in gene expression was persistently observed using different mRNAs (Figures 1, 2, 3, 4). Most importantly, the use of RTEm26-CTE has another great advantage, because this combination avoids the presence of adjacent repeated sequences, which may cause plasmid instability during growth in bacteria.

## Discussion

The rather unexpected finding of this work was that the combination of two retroviral/retroelement-derived *cis-*acting RNA export elements, RTE and CTE, synergistically increased expression of different retroviral mRNAs that are otherwise poorly expressed (Figures 1, 2, 3, 4). Since the function of RTEm26-CTE is conserved in mammalian cells, their integration into expression vectors provides a potent tool to improve expression of poorly expressed, unstable retroviral mRNAs to levels otherwise only achieved via more cumbersome RNA optimization.

Whereas the main restriction retroviral mRNAs encounter is their nucleocytoplasmic transport, other mRNAs may have different restrictions. The question arises whether the RTEm26-CTE combination has any effect on the expression of genes or cDNAs, which have posttranscriptional restrictions other than those of the lentiviral mRNAs. No improvement of expression was found using either the RTE, the CTE or the RTEm26-CTE combination in a MuLV-derived retroviral vector [37], while insertion of the woodchuck element WPRE augmented expression of this MuLV mRNA. These data indicate that oncoretrovirus and lentivirus expression have distinct restrictions. We further tested whether the presence of these RNA export elements could counteract posttranscriptional control that is not exerted at the RNA transport level but only involves cytoplasmic control. We noted that these export elements, as expected, did not alleviate the downregulatory effect of the AU-rich element (ARE)-containing IL-3 mRNA using the GFP-IL-3 hybrid mRNAs as a model system [38]. Thus, this finding supports the specific mechanism of function of the RTE, CTE and RTEm26-CTE, namely nucleocytoplasmic export. For this reason, we tested RTEm26-CTE effect upon insertion into some of our already RNA-optimized HIV and SIV *gag *and *env *cDNAs vectors, whose mRNAs are efficiently exported leading to very high expression in cultured cells. As expected, we only found a less than 2-fold effect on this already optimized mRNAs. It remains to be tested whether export signals like the RTEm26-CTE could act as additional positive acting signals and mediate higher expression levels in primary cells, for example upon DNA vaccination of animals or using gene transfer vectors.

NXF1 provides a key molecular link between mRNA and components of the nuclear pore complex. A possible model to explain the synergistic effect of RTE and CTE is that the duplication of these export elements may provide an improved target for NXF1 resulting in more efficient nucleocytoplasmic mRNA transport. Using *in vitro *gel-shift assays, we found that the binding of NXF1 (aa 61–372) to radiolabeled CTE is competed similarly by both excess cold CTE as well as RTEm26-CTE RNAs (data not shown). These data indicate that NXF1 binds to CTE as well as to the RTEm26-CTE RNA targets with similar affinity. We have previously shown that NXF1 is not a high affinity binder of RTE when compared to the CTE [6], suggesting the role of a distinct cellular protein mediating RTE RNA export. It is plausible that this putative factor tethers the RTE-RNAs directly or indirectly to the NXF1 pathway. Therefore, it is likely that the putative RTE-binding protein and NXF1 may act cooperatively. Studies are on-going to delineate the detailed mechanism of function mediating this cooperativity.

mRNA expression is controlled at several steps including nuclear export, cytoplasmic trafficking and polysomal association. The use of strong mRNA export elements is a powerful tool to alleviate restrictions linked to nuclear export. For a subset of lentiviral mRNAs encoding *gag*, *pol *or *env*, posttranscriptional control has been shown at multiple steps of export and polysomal association. The presence of potent RNA export elements is sufficient to alleviate all of theses restrictions. Integration of RTEm26-CTE into lentiviral vectors will increase gene expression essential for applications such as in gene therapy that are otherwise only obtained through RNA optimization. For DNA-based vaccine vectors, it remains to be seen whether the presence of strong binding sites for the cellular mRNA transport machinery is of further advantage when introduced into primary tissues in animals as compared to cultured cells. In addition, these retroviral/retroelement derived RNA export elements provide unique tools to further dissect mechanisms involved in posttranscriptional regulation of viral and cellular genes.

## Conclusion

The use of the combination of RNA export elements, comprising the up-regulatory mutant RTEm26 and the CTE, potently increase lentiviral gene expression.

## Methods

### Plasmids

The RTE, RTEm26, and CTE were inserted into pNLgag [23,24,39] between the *gag *gene and the 3'LTR and have been described [7]. RTE or RTEm26 was inserted into the *SacII *site located 5' to the CTE, generating pNLgagRTE-CTE and pNLgagRTEm26-CTE, respectively. In pNLgag RTEm26-SP1-CTE, a spacer sequence (SP1) of 325 nt from a synthetic HIV-1 *tat *gene (*Bam*HI-*Xba*I from plasmid 32H) was inserted between RTEm26 and CTE. In pNLgag RTEm26-SP2-CTE, a spacer (SP2) from a different source (*cat *gene) of 325 nt was inserted. Similarly spacers or 202 and 100 nt were inserted. The bovine growth hormone polyadenylation signal was inserted between *Sal*I and *Xho*I sites 3' to RTEm26-CTE replacing the 3'LTR. pNLcgag [24] is similar to pNLgag, except it lacks the major splice donor. pNL1.5E expresses the authentic HIV-1 env cDNA from the LTR promoter [40]. RTE, CTE and RTEm26-CTE were inserted as *Sma*I-*Xho*I fragment between the *env *gene and the 3' LTR into *Blp*I and *Xho*I digested pNL1.5E. The Rev-independent clones of NL4-3 [RRE(-)Rev(-), RRE(-)Rev(-)CTE, and RRE(-)Rev(-)RTE] have been published previously [6,12,41]. RTEm26-CTE was inserted into the *Xho*I site of the RRE(-)Rev(-) NL4-3. The SIVmac239 RRE(-)Rev(-)nefdelCTE is similar to the published SIVmac239 RRE(-)Rev(-)Nef(-)CTE [35] but contains an additional deletion of the remaining *nef *region 3' to the CTE [42]. RTEm26-CTE was inserted in the place of CTE. The GFP-IL-3 plasmid contains the IL3 3'UTR inserted 3' to the enhanced green fluorescent protein (GFP) gene in pFRED25 [43]. RTEm26, CTE, or RTEM26CTE were inserted between GFP and the 3'UTR. These elements were further inserted between the cDNAs and the polyadenylation signals of vectors expressing the RNA-optimized HIV-1 *env *(75 H).

### Transfections

Human HLtat, a HeLa-derivative producing HIV *tat *[44] or human 293 cells were transfected with 1 μg of the NLgag plasmids. HLtat provides Tat to activate gene expression from the viral LTR promoter. For transfection of 293 cells a *tat *expression plasmid, pBstat, was also co-transfected. We routinely analyzed 2–3 independent clones in duplicate determinations. Two to three days later, the cell extracts were analyzed for Gag expression using a commercial HIV-1 p24gag or the SIV p27gag antigen capture assay. Gag and Env production was also analyzed on Western immunoblot using plasma from HIV-1 infected persons, rabbit anti-HIV-1 env serum or SIVmac infected rhesus macaques [23]. Cotransfection of 0.8 μg of the GFP expression vector pFRED25 [43] served as internal control. Cotransfection of the secreted version of alkaline phosphatase SEAP [45] as internal control was used in some experiments and SEAP levels were determined from the culture supernatant using a commercial kit (Tropix, Inc.). Transfections of 293 cells were performed using FUGENE-6, whereas the Calcium-phosphate coprecipitation technique was used for HeLa cells. GFP-IL3 plasmids were transfected into NIH3T3 cells and analyzed by fluorescent activated cell sorting (FACS) as described [38]. Total and cytoplasmic polyadenylated mRNA was isolated and analyzed as described [12,46]. Hybridization of the blots with a GFP probe was used to evaluate transfection and RNA extraction efficiency. Blots were quantitated using the STORM860 phosphoimager.

## Abbreviations

CTE, constitutive Transport Element; RTE, RNA Transport Element; RRE, Rev-Responsive Element; HIV-1, human immunodeficiency virus type 1; SIV, simian immunodeficiency virus; IAP, intracisternal A-particle retroelement; SRV/D, simian type D retroviruses; NXF1, nuclear export factor 1.

## Competing interests

The author(s) declare that they have no competing interests.

## Authors' contributions

SS generated RTEM26-CTE constructs and performed expression studies; RJ, MR, AvG provided additional constructs and performed expression studies; DM performed in vitro binding studies; JB, CA performed experiments in using infectious HIV and SIV and provided technical assistance; ASZ and CM provided reagents and intellectual input; GNP provided intellectual input and contributed to the manuscript; BKF directed the project and wrote the manuscript.
